# Development and Characterization of Small Molecule Chemical Probes for Alzheimer's Disease‐associated Human RNA Helicases

**DOI:** 10.1002/alz70859_096394

**Published:** 2025-12-25

**Authors:** U Hang Chan, Fengling Li, Frances M. Bashore, Scott Houliston, Catherine Vu, Irene Chau, Alison D. Axtman, Levon Halabelian

**Affiliations:** ^1^ University of Toronto, Toronto, ON Canada; ^2^ Structural Genomics Consortium, Toronto, ON Canada; ^3^ Structural Genomics Consortium, Chapel Hill, NC USA; ^4^ University of North Carolina at Chapel Hill, Chapel Hill, NC USA; ^5^ University Health Network, Toronto, ON Canada

## Abstract

**Background:**

To diversify Alzheimer’s Disease (AD) drug targets, a bioinformatics core is established to provide an unbiased ranking of AD risk‐associated genes by integrating multiple lines of genetic and multi‐omic evidence. From which, several RNA helicases, including RIG‐I‐like receptor 3 (LGP2), melanoma differentiation‐associated protein 5 (MDA5) and Dead Box 1 (DDX1) have been identified as high priority targets differentially expressed in AD brains. All three helicases play a role in the innate immune response pathway against viral RNA. Given the previous link between viral infection and AD pathology, this prompted the development of small molecule chemical probe against these targets to further elucidate their roles in AD.

**Method:**

Purified proteins were used for ATPase assay development and compound screening. The ATPase assay was performed in the presence of annealed 24mer RNA, double‐stranded RNA (dsRNA) with a 25‐nt 3ʹ overhang, or single‐stranded DNA (ssDNA). We employed DNA‐encoded chemical library (DEL) and computational methods for small molecule hit discovery. Hit confirmation was carried out by ATPase assay, Surface Plasmon Resonance (SPR), Differential Scanning Fluorimetry (DSF) and 19Fluorine‐ Nuclear Magnetic Resonance (19F‐NMR). Hit expansion was carried out for the most promising hits to increase potency and selectivity.

**Result:**

We describe the development and optimization of a bioluminescence assay to kinetically characterize the activity of three human RNA helicases involved in innate immune response pathway, including MDA5, LGP2, and DDX1. Through DEL‐ML screening, we identified a selective hit for MDA5, and characterized its activity by ATPase assay with IC50 of 8 µM, and orthogonally confirmed by F‐NMR. Ongoing studies aim to elucidate the ligand binding site using X‐ray crystallography.

**Conclusion:**

We present a robust high‐throughput in vitro assay designed for small molecule screening in a 384‐well format, enabling hit optimization and facilitating the discovery of inhibitors for MDA5, LGP2, and DDX1. Through DEL‐ML screen, we identified a selective MDA5 inhibitor that can be used to further interrogate its role in AD pathogenesis, and serve as a chemical starting point for future drug discovery efforts. This ligand represents first‐in‐class small molecule inhibitor for MDA5, a target that has been underexplored in the context of its role in AD.

